# Health care expenditure associated with overweight/obesity: a study among urban married women in Delhi, India

**DOI:** 10.18203/2394-6040.ijcmph20150488

**Published:** 2015-08

**Authors:** Praween Agrawal, Sutapa Agrawal

**Affiliations:** 1 UNICEF India Country Office, New Delhi, India; 2 Centre for Control of Chronic Conditions, Public Health Foundation of India, India

**Keywords:** Obesity, Health expenditure, Body mass index, NFHS-2 follow-up survey, Multiple logistic regressions

## Abstract

**Background:**

Obesity is a multifaceted problem with wide-reaching medical, social and economic consequences. While health consequences are much known, but due to paucity of data, economic consequences are less known in India. The prevalence for excessive weight particularly among women population has been increasing dramatically in India in the last decades. We examined the economic burden on individual and households due to overweight and obesity among women in the national capital territory of India, Delhi. We particularly examined the health expenditure pattern in absolute amount as well as a proportion to their household expenditure among women according to their level of body mass index (BMI).

**Methods:**

A population based follow-up survey of 325 ever-married women aged 20-54 years residing in the national capital territory of Delhi in India, systematically selected from the second round of National Family Health Survey (NFHS-2, 1998-99) samples who were re-interviewed after four years in 2003. Women’s expenditure on health has been seen as a gross and as a ratio of total household expenditure. Anthropometric measurements were obtained from women to compute their current body mass index. Multiple logistic regression analysis was used to estimate the odds ratios adjusting for various socio demographic confounders.

**Results:**

A significantly (p<0.0001) higher monthly gross health expenditure as well as proportion of total household expenditure was found according to the women’s level of BMI. Average monthly health expenditure was Rs. 132 among overweight women, Rs 143 among obese women which further increased to Rs. 224 among morbidly obese women compared to only Rs 68 among normal weight women. Almost, 15% overweight, 16% obese and 21% morbidly obese women (p<0.0001) had economic burden which accounts for more than 5% of their total household expenditure on their health compared to only 10% normal weight women. Significantly, obese and morbidly obese women were more than two times more likely to spend higher amount on their health (OR 2.29 95% CI: 1.07-4.90; p=0.033) than normal weight women. Also overweight women were significantly two times more likely to spend high proportion on their health with respect to total household expenditure (OR 2.11; 95% CI: 1.03-4.35; p=0.042) than normal weight women.

**Conclusions:**

There is substantial economic burden of obesity for individuals as well as for the households which calls for urgent intervention in the obesity awareness and health promotion among Indian women who faced the greatest burden of increasing body weight in the last decade. Prevention is obviously more cost effective than treatment, both in terms of healthcare and personal costs. Health care providers and policy makers need to critically understand the issue of obesity and develop effective policies and programs for its prevention among Indian women.

## Introduction

Strengthened by the forces of globalization—including increased amounts of international trade, travel, and shared communication—the obesity epidemic is rapidly becoming a worldwide problem.^1^ The obesity epidemic is spreading widely to low-income and middle-income countries as a result of new dietary habits and sedentary ways of life, fuelling chronic diseases and premature mortality.^2^ Before 1980, obesity rates were generally much lower than 10%. Since then, rates have doubled or tripled in many countries, including the low and middle income countries. Smaller increases in overweight were recorded in India (rates for women rose from 10.6% to 12.6% between 1998–99 and 2005–06), but increases were steepest in urban areas in the west of the continent, where rates approached 40% in the early 2000s, almost doubling in less than 10 years.^3^–^4^ India has more than 30 million obese people, and the number is increasing alarmingly.^4^–^5^ The problem is more acute among women than men.^6^ In urban India, more than 23% of women are either overweight or obese, which is higher than the prevalence among men (20%).^3^

Obesity is a multifaceted problem with wide-reaching medical, social and economic consequences. For the obese person, excess weight denotes an increased risk of disabling chronic diseases, lowered quality of life and loss of earnings since obesity-related health burdens carry staggering financial implications.^7^ It has been estimated that obesity accounts for 2% to 7% of total healthcare costs. There are also other costs to consider such as reduced quality of life and productivity loss attributed to medical leave.^8^ The lifetime medical expenditure for an obese person aged 20 year old ranges from $5,340 to $29,460, increasing proportionally with a rising Body Mass Index (BMI).^9^ For society, obesity is a major economic burden and treatment costs of diseases directly attributable to obesity are estimated to correspond to about 2-10% of the total health care expenditure. The indirect costs arising from loss of productivity due to obesity are even higher.^10^ Obese people spend 42 percent more on healthcare costs than healthy weight people.^9^ India’s rapid increase in diet-related non-communicable diseases and their costs project similar economic cost of under nutrition and overnutrition.^11^

Often overshadowed by the health and social consequences of obesity is the economic cost to society and to the individual. Obesity is expensive, and a burden on health systems. Throughout their lives, health care expenditures for obese people are at least 25% higher than for someone of normal weight and increase rapidly as people get fatter. The economic costs of obesity rise with the increasing BMI due to the escalating risk, prevalence and morbidity of co-morbid disease.^11^ In general, disease prevalence and total direct medical costs increase as patients gain weight. Visits to the clinicians increase significantly with obesity. Several studies conducted in developed countries^9^,^12^–^16^ reported that morbidly obese persons (BMI of 35 or higher) have a 24% higher rate of outpatient visits and 74% more hospital days than those with a BMI below 30. It is noted that social trends responsible for the obesity epidemic that pervades our society and affects all of us and the economic cost of obesity are born by everyone.^17^

Most available recent data from India showed overweight and obesity together among women is 12.6% and almost similar percentage of underweight and overweight women coexists in urban India (25% underweight and 23.5% overweight or obese).^3^ In the light of the increases in population weights in India, it is worthwhile to examine the economic consequences of excess weight gain in terms of individual’s expenditure on health care more specifically among adult women in India who are the sufferer of largest weight gain as compared to men.^18^,^19^ In this study, we examined the economic burden due to overweight and obesity among women in India. We particularly examined the health expenditure pattern in absolute amount as well as proportion to their household expenditure for women according to their level of BMI.

## Methods

### Study location and population

The present paper utilises data collected for the Doctoral dissertation by the first author.^21^ Full details of the study have been presented elsewhere.^20^,^21^ Briefly, during May-June 2003, a follow up survey was carried out in the national capital territory of Delhi using the same sample derived from the National Family Health Survey-2 (NFHS-2) conducted during 1998-99. Delhi which has a heterogeneous, multicultural population representative of the Indian urban scenario was chosen as the preferred location for this study. NFHS-2 collected demographic, socio-economic, and health information from a nationally representative sample of 90,303 ever-married women aged 15-49 years in all states of India (except the union territories) covering more than 99% of the country’s population with a response rate of 98%. Details of sample design, including sampling frame are provided in the national survey report.^5^

From the 1998-99 NFHS-2 Delhi samples, 325 women aged 15-49 years chosen systematically, were re-interviewed in a follow up survey after four years in 2003 using an interview schedule. Their weights and heights were again recorded in the follow-up study by the researcher (using the same equipment used in NFHS-2) to compute their current body mass index. In addition to these measurements, detailed information was collected on their dietary habits, lifestyle behaviour, psycho-social problems (asked only to overweight and obese women only) along with other socio-demographic characteristics. Self-reported information on woman’s day to day problems, body image dissatisfaction, sexual dissatisfaction and stigma and discrimination which are the main response variable in this study were sought through face-to-face interview with a interview schedule.

### Sample Selection, response rate and sample size

Earlier studies on obesity in India and other developing countries have shown that overweight and obesity are predominant in urban areas and among women.^22^ Therefore, only urban Primary Sampling Units (PSUs) were chosen for the follow-up survey in Delhi. The sample frame for the follow up survey was fixed to include women in all BMI categories and literacy levels. The aim was to have a sample size of at least 300 women, 100 from each of the three BMI categories (normal, overweight, and obese). At the time of revisit, several issues such as migration, change of address, non-response and non-availability of respondents tend to reduce the desired sample size. Potential loss during follow-up^23^–^24^ was dealt with increasing the initial sample size (double than required) to get the desired sample size for the study.

**Figure 1:**
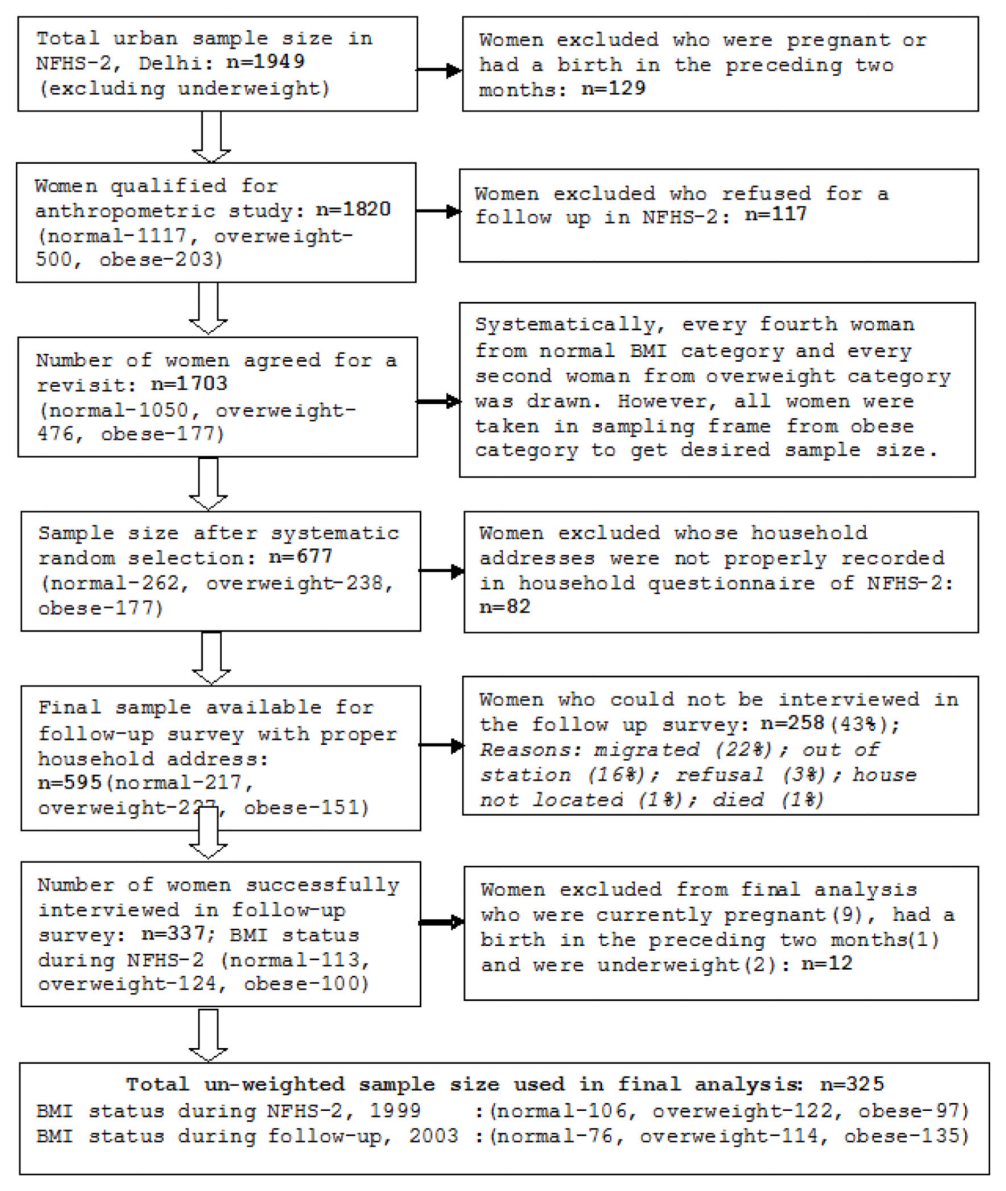
Selection of sample in the follow-up survey and response rate.

In NFHS-2 Delhi sample, 1117, 500, and 203 women were normal, overweight and obese respectively. In NFHS-2 survey questionnaire respondents were asked, ‘*Would you mind if we come again for a similar study at some future date after a year or so?*’ Those women who objected for a revisit were excluded from the follow up survey, and thus there remained 1050 normal, 476 overweight, and 177 obese women in the sampling frame. Samples were drawn from each of these three categories through systematic stratified random selection using a random number. From the normal BMI category, every fourth woman and from the overweight category every second woman was drawn. In the obese category all women were included in the sample to get the desired sample size. This resulted into selection of a total of 677 women–262 normal, 238 overweight and 177 obese. For the follow up survey, the addresses of the selected women were obtained from the NFHS-2 Household Questionnaires. Sample size was further reduced due to non-availability of some questionnaires and non-identified addresses. Finally, a total of 595 women–217 normal, 227 overweight and 151 obese were selected for the follow up interview. Details of the sample selection and response rate are illustrated in the schematic diagram (Figure 1).

In the follow-up survey, 57% of the eligible samples (337 women) were successfully interviewed–113 normal, 124 overweight and 100 obese women. 43% of the sample (258 women) could not be interviewed as they were out of station (16%), had migrated (22%), their residence was un-located (1%), died (1%) or refused for an interview (3%). Women who were pregnant (n=9) at the time of the follow-up survey, women who had given birth during the two months preceding the survey (n=2) and underweight women (n=1) have been excluded from the final analysis. Therefore, the findings are based on the remaining 325 respondents of the follow up survey. A separate analysis using NFHS-2 data shows that the socio-demographic characteristics of those interviewed and those could not be interviewed in the follow up survey were similar (data not shown) indicating that the follow-up sample appears representative of the NFHS-2 sample population.

### Anthropometric measurements

In NFHS-2 (executed by the field investigators) as well as in the follow-up survey (executed by the researcher), each ever-married woman was weighed in light clothes with shoes off using a solar-powered digital scale with an accuracy of ±100 gms. Their height was also measured using an adjustable wooden measuring board, specifically designed to provide accurate measurements (to the nearest 0.1 cm) in a developing country field situation. These data were used to calculate their individual BMIs. Practical and clinical definitions of overweight and obesity are based on the BMI, which is computed by dividing weight (in kilogram) by the square of height (in meter) [kg/m2].^23^ A woman with a BMI between 25 and 30 is considered to be overweight, a BMI of greater than 30 is considered to be obese. A woman with a BMI between 18.5 and 24.9 is considered normal, and if the BMI is below 18.5 the woman is considered to be underweight.^23^

### Variables studied

We assessed the response to the question on “How much is the average expenditure of your family (in Indian rupees) in different items, such as food, house rent, clothing, education, health, entertainment and other, to analyze the health expenditure pattern in the study. Women’s health expenditure has been computed in terms of gross and as a proportion of total household expenditure.

Characteristics of the respondents that are included as potential confounders in the study are: age groups (20-34, 35-54, education (illiterate, literate but <middle school complete, middle school complete, high school complete and above), religion (Hindu, Muslims, Sikh and Others), caste/tribe (Scheduled caste/tribes, Other backward class, Others), household standard of living (low/medium, high), employment status (not working, working), and media exposure (never reads newspaper, reads newspaper occasionally, reads newspaper daily). For a full definition of variables see Table 1.

### Statistical methods

Data are analyzed using descriptive statistics. The association between overweight/obesity and health expenditure was estimated using multivariate logistic regression models after controlling for socio-economic and demographic factors and examining for the independent effects of the covariates. All analysis was done using SPSS Version 19 (IBM SPSS Statistics, Chicago, IL, USA).

### Ethical approval

The study received approval from the International Institute for Population Science’s Research Committee. Informed consent was obtained from all respondents in both NFHS-2 and the follow-up survey before asking questions and before obtaining measurements of their height and weight. The analysis presented in this study is based on secondary analysis of the survey data with all identifying information removed.

## Results

Table 1 presents the characteristics of the study population. In the study sample, there were almost one-fourth normal weight women, more than one-third were overweight (35.2%), 29% obese and 13% were morbidly obese. Two-fifths respondents were below 35 years and 60% were over 35 years of age, the mean age of the respondents being 41.2 years. Over half the study population (58%) had completed high school education while one-seventh was illiterate. Almost 80% of the respondents were Hindu, the rest being Muslim, Sikh and Others. Regarding caste/tribe distribution, ‘Others’ were predominant (77%). Majority of the respondents (82%) belonged to households with a higher standard of living (SLI) whereas 18% women belonged to households with a medium or lower SLI. A majority of the women (91%) were not working.

Table 2 presents gross monthly health expenditure and proportion of heath expenditure to the total household expenditure among normal weight, overweight, obese and morbidly obese women. Significantly higher monthly gross health expenditure was found according to the women’s level of BMI. Average monthly health expenditure was Rs. 132 among overweight women, Rs. 143 among obese women which further increased to Rs. 224 among morbidly obese women compared to only Rs. 68 among normal weight women. About 28% overweight women spent more than Rs. 100 per month on their health compared to 35% obese women and almost half of the morbidly obese women compared to only 10% normal weight women. However, data shows that a significant higher proportion of normal weight women (64%) spent nothing on their health compared to 60% overweight women, 45% obese women and only 34% morbidly obese women.

The ratio of health expenditure of women to the total household expenditure was also higher as per their level of BMI. Women with a higher BMI had higher economic burden. Average health expenditure of women to the total household expenditure was 3.3 % among overweight women, 2.5% among obese women which further increased to 5.1% among morbidly obese women compared to only 1.6% among normal weight women. Further, 15% overweight, 16% obese women and 21% morbidly obese women had economic burden of more than 5% expenditure on their health of their total household expenditure compared to 10% normal weight women.

Table 3 presents the percentage of women who reported high monthly health expenditure and high proportion of heath expenditure to the total household expenditure by their level of BMI and background characteristics. Almost 28% overweight women, 33% obese women and 47% morbidly obese women reported high monthly health expenditure compared to only 16% normal weight women. High monthly health expenditure was reported significantly higher among women aged 35-54 years (33%) compared to women aged 20-34 years (16%). However, by education, caste and standard of living (SLI) no differential was noticed.

When health expenditure was seen as ratio of health expenditure of women to the total household expenditure as economic burden, SLI was found significant along with age. A significant higher proportion of women from low/medium SLI (39%) were spending higher proportion on their heath to the total household expenditure than women from high SLI (22%). Other characteristics were found insignificant. Table 4 provides the results of logistic regression (Odds ratio with 95% confidence interval (CI) for the determinants of women’s high gross health expenditure and high proportion of health expenditure in total household expenditure in two different models after adjusting for socio-economic demographic characteristics.

The result shows that obese and morbidly women were more than two times significantly likely to spend higher amount on their health (OR 2.29 95% CI: 1.07-4.90; p=0.033) than normal weight women. Also overweight women were 1.6 times more likely to spend higher amount on their health (OR 1.64 95% CI: 0.81-3.34; p=0.169) than normal weight women but result was not significant. Other socio-economic demographic characteristics such as education, caste, religion, SLI, employment and media exposure were found insignificant to spend higher on their health except their age. Women aged 35-54 years were almost two and half times more likely to spend higher amount on their health (OR 2.41 95% CI: 1.35-4.31; p=0.003) than women aged 20-34 years.

Looking into the high proportion of health expenditure in total household expenditure, overweight women were significantly two times more likely to spend high proportion on their health to the total household expenditure (OR 2.11; 95% CI: 1.03-4.35; p=0.042) than normal weight women. Also obese and morbidly women were about two times more likely to spend high proportion on their health to the total household expenditure (OR 2.11; 95% CI: 0.94-4.76; p=0.071) than normal weight women but result was not significant. Other socio-economic demographic characteristics of women were not found significant in spending high proportion on their health to the total household expenditure except their age and SLI. Women aged 35-54 years were close to three times more likely to spend high proportion on their health to the total household expenditure (OR 2.83 95% CI: 1.53-5.24;p=0.001) than women aged 20-34 years. Women from high SLI were almost half times less likely to spend high proportion on their health to the total household expenditure (OR 0.41 95% CI: 0.20-0.836; p=0.018) than women from low/medium SLI.

## Discussion

In this study, we aimed to estimate the health care expenditure associated with overweight and obesity among urban married women in Delhi, India. Our study found that average monthly health expenditure and as well as proportion health expenditure to total household expenditure significantly increased with level of BMI of women. Obese and morbidly obese women were more than two times significantly more likely to spend higher on their health than normal weight women. Also overweight women were significantly two times more likely to spend high on their health proportion to the total household expenditure. Women belonging to lower socio economic status were the most affected and spend significantly higher on their individual health as proportion to the total household expenditure. Also middle aged and older women were spending almost double on their health than younger women.

Finding of this study has great importance as several studies have shown obesity in India is on rise particularly among women population^18^ and in urban India.^6^ The level of obesity is significantly associated with economic burden as health expenditure among urban Indian women. Proportion of health expenditure to the total household expenditure among poor was higher. The rising level of obesity in India will increase in the health expenditure burden in general and among poor in particular.

Obesity is estimated to be responsible for 1% to 3% of total health expenditure in most countries (5% to 10% in the United States) and costs will rise rapidly in coming years as obesity-related diseases set in.^14^ Our study also found that obese women have 2% to 3% more expenditure on health to the total household expenditure. Throughout their lives, health care expenditures for obese people are at least 25% higher than for someone of normal weight and increase rapidly as people get fatter.^7^ Obesity is thus expensive and a burden on health systems as well as individuals.

Some strengths of our study deserve comment. First, our study is based in the national capital territory of Delhi which typifies a multicultural and multiethnic population representing India’s growing urban scenario. Second, there is dearth of studies in India which examine the individual and household expenditure on health due to overweight/obesity among Indian women by analyzing representative data on anthropometric measures, which is exceptional in India. Our study used actual measured weights and heights without relying on self-reported values for these measures, which could otherwise be over- or underestimated. For these reasons our study is an important contribution to address this existing gap in knowledge in India. Fourth, in this study we have considered ever-married adult women to examine the health consequences of BMI. The reason for this is that NFHS-2 confirmed the marked rural–urban differences in prevalence of obesity among women, and also we could find a rising trend of obesity among married women between the second^5^ and third NFHS.^3^

Some limitations of this study also deserve attention. The poor response rate was a limitation in this study because it could have introduced participation bias, resulting in inaccuracy and lack of generalizability of the results. Although rigorous methods were used, including cross checks and back-checks, to achieve high quality data, some measurement errors cannot be ruled out.^5^ Secondly, although we adjusted for several key socio-demographic factors, other potentially confounding characteristics and behaviors that were not measured in our study may have confounded our results. Furthermore, because of the cross-sectional nature of our study and the interdependency of many of the variables, no definite statement on temporality or causal directions among the independent and dependent variables can be made. Lastly, we did not use any standard instruments for collecting data on the variables studied, and thus the questions were developed for this study by the authors. This could have resulted in misclassification of information and makes the information non-comparable to other studies that did use standard instruments.

## Conclusion

There is substantial economic burden of obesity for individuals as well as for the households which calls for urgent intervention in the obesity awareness and health promotion among Indian women who bared the greatest burden of increasing body weight in the last decade. Prevention is obviously more cost effective than treatment, both in terms of healthcare and personal costs. Health care providers and policy makers need to critically understand the issue of obesity and develop effective policies and programs for its prevention among Indian women. A timely prevention will reduce the health care expenditure burden of much chronic co-morbidity, like diabetes, cardiovascular diseases, hypertension, and infertility^25^ which are associated with obesity. This can be achieved either through undertaking separate urban health programme or incorporating special clause in the proposed National Urban Health Program, citing the importance of healthy diet and physical exercise among women in India.

## Figures and Tables

**Table 1:** Selection of sample in the follow-up survey and response rate.

Characteristics	Percent	Number of women
**Current Body Mass Index (BMI)^1^**		
Normal (BMI 18.50-24.99 kg/m^2^)	23.1	75
Overweight (BMI 25.0-29.99 kg/m^2^)	35.2	114
Obese (BMI ≥30.0-34.99 kg/m^2^)	29.0	94
Morbidly Obese (BMI ≥35.0 kg/m^2^)	12.7	41
**Age**		
20-34	40.1	130
35-54	59.9	194
Mean age [±SD]	41.2[±11.2]	324
**Education^2^**		
Illiterate	17.9	58
Literate, <middle school complete	17.3	56
Middle school complete	14.5	47
High school complete and above	50.3	163
**Religion**		
Hindu	81.2	263
Muslim	8.0	26
Sikh and Others^3^	10.8	35
**Caste/tribe^4^**		
Scheduled caste/tribes	13.7	44
Other backward class	9.0	29
Others	77.3	249
**Standard of living index^5^**		
Low/Medium	17.9	57
High	82.1	261
**Employment status**		
Not working	91.3	295
Working	8.7	28
**Media Exposure**		
Never reads newspapers	46.0	149
Reads newspapers occasionally	25.3	82
Reads newspapers daily	28.7	93
***Number of women***	100.0	324
**Media Exposure**		
Never reads newspapers	46.0	149
Reads newspapers occasionally	25.3	82
Reads newspapers daily	28.7	93
***Number of women***	100.0	324

1 Note: Women who were pregnant at the time of the survey, or who had given birth during the two months preceding the survey, were excluded from these anthropometric measurements.

2 Illiterate-0 years of education, literate but less than middle school complete-1–5 years of education, middle school complete-6–8 years of education, high school complete or more-9+ years of education

3 Buddhist, Christian, Jain, Jewish, Zoroastrian

4 Scheduled castes and Scheduled tribes are identified by the Government of India as socially and economically backward and needing protection from social injustice and exploitation; Other Backward class category is a diverse collection of intermediate castes that were considered low in the traditional caste hierarchy but are clearly above SC; Others’ is a default residual group that enjoys higher status in the caste hierarchy.

5 Standard of living (SLI) was defined in terms of household assets and material possessions and these have been shown to be reliable and valid measures of household material well-being. It is an index which is based on ownership of a number of different consumer durables and other household items. It is calculated by adding the following scores: house type: 4 for pucca, 2 for semi pucca, 0 for kachha; toilet facility: 4 for own flush toilet, 2 for public or shared flush toilet or own pit toilet, 1 for shared or public pit toilet, 0 for no facility; source of lighting: 2 for electricity, 1 for kerosene, gas or oil, 0 for other source of lighting; main fuel for cooking: 2 for electricity, liquefied natural gas, or biogas, 1 for coal, charcoal, or kerosene, 0 for other fuel; source of drinking water: 2 for pipe, hand pump, or well in residence/yard/plot, 1 for public tap, hand pump, or well, 0 for other water source; separate room for cooking: 1 for yes, 0 for no; ownership of house: 2 for yes, 0 for no; ownership of agricultural land: 4 for 5 acres or more, 3 for 2.0-4.9 acres, 2 for less than 2 acres or acreage not known, 0 for no agricultural land; ownership of irrigated land: 2 if household owns at least some irrigated land, 0 for no irrigated land; ownership of livestock: 2 if own livestock, 0 if not own livestock; durable goods ownership: 4 for a car or tractor, 3 each for a moped/scooter/motorcycle, telephone, refrigerator, or colour television, 2 each for a bicycle, electric fan, radio/transistor, sewing machine, black and white television, water pump, bullock cart, or thresher, 1 each for a mattress, pressure cooker, chair, cot/bed, table, or clock/watch. Index scores range from 0-14 for low SLI to 15-24 for medium SLI to 25-67 for high SLI.

**Table 2:** Monthly expenditure pattern on health among normal weight, overweight, obese and morbidly obese women age 20-53 years, Delhi.

Health expenditure	Current BMI				
	Normal	Overweight	Obese	Morbidly Obese	Chi sq. p value
Individual monthly health expenditure**					0.012
Nil	64.4	59.6	44.7	34.2	
Up to Rs.100	16.4	12.3	20.2	18.4	
More than Rs.100	19.2	28.1	35.1	47.7	
Average [±SD] monthly expenditure in Rs	68.0[±133.4]	132.7[±234.4]	143.7[±204.9]	224.8[±370.9]	
**Percent on health expenditure out of total household expenditure**					0.354
Nil	70.7	66.3	59.5	54.5	
Up to 5%	19.0	15.4	28.6	27.3	
More than 5 %	10.3	14.9	15.7	21.4	

**Table 3:** Percentage of women reported high monthly health expenditure (above mean) and high proportion of health expenditure (above mean) in total household expenditure according to level of their BMI and background characteristics, Delhi.

Characteristics	High (above mean) monthly expenditure on women’s health	χ^2^ p values	High (above mean) proportion of health expenditure out of total household expenditure	χ^2^ p values
**Current BMI**		0.008		0.300
Normal	16.3		20.0	
Overweight	27.5		28.3	
Obese	33.3		32.0	
Morbidly Obese	47.1		30.8	
**Age**		<0.0001		<0.0001
20-34	16.4		16.7	
35-54	33.1		34.2	
**Education**		0.997		0.097
Illiterate	23.0		36.1	
Literate, <middle school complete	24.6		26.3	
Middle school complete	24.5		25.5	
High school complete and above	24.8		20.1	
**Religion**		0.545		0.441
Hindu	23.5		24.1	
Muslim	36.4		36.8	
Sikh and Others	27.3		29.0	
**Caste/tribes**		0.759		0.191
Scheduled caste/tribes	22.1		32.1	
Other backward class	27.3		35.5	
Others	25.9		23.0	
**Standard of living index**		0.229		0.007
Low/ Medium	20.6		39.3	
High	26.0		22.3	
**Working status**		0.071		0.477
Not working	23.9		25.8	
Working	40.0		22.7	
**Media exposure**		0.359		0.234
Never reads newspapers	24.5		29.4	
Reads newspapers occasionally	20.7		21.8	
Reads newspapers daily	30.8		20.8	
**Total**	24.8		25.4

**Table 4:** Adjusted effects (Odds ratio with 95% confidence interval (CI) of level of BMI on women’s high health expenditure and high proportion of health expenditure out of total household expenditure, Delhi.

Characteristics	High monthly health expenditure (above mean)	P values	High monthly health expenditure (above mean) proportion to the total household expenditure	P values
	OR [95% CI]		OR [95% CI]	
**Current BMI**				
Normal ^R^	1		1	
Overweight	1.64[0.81-3.34]	0.169	2.11[1.03-4.35]	0.042
Obese & Morbidly obese	2.29[1.07-4.90]	0.033	2.11[0.94-4.76]	0.071
**Age**				
20-34 ^R^	1		1	
35-54	2.41[1.35-4.31]	0.003	2.83[1.53-5.24]	0.001
**Education**				
Illiterate ^R^	1		1	
Literate, <middle school complete	1.23[0.48-3.13]	0.667	0.93[0.37-2.36]	0.881
Middle school complete	1.11[0.40-3.10]	0.844	0.97[0.35-2.72]	0.951
High school complete and above	0.95[0.34-2.66]	0.922	0.68[0.24-1.97]	0.478
**Religion**				
Hindu ^R^	1		1	
Muslim	2.30[0.83-6.35]	0.108	1.42[0.47-4.30]	0.541
Sikh and Others	1.29[0.52-3.20]	0.585	1.76[0.70-4.44]	0.231
**Caste/tribes**				
Scheduled caste/tribes ^R^	1		1	
Other backward class	0.81[0.27-2.43]	0.611	0.82[0.29-2.35]	0.711
Others	0.75[0.32-1.77]	0.245	0.53[0.23-1.24]	0.142
**Standard of living index**				
Low/ Medium^R^	1		1	
High	1.26[0.58-2.74]	0.567	0.41[0.20-0.86]	0.018
**Employment status**				
Not working ^R^	1		1	
Working	1.92[0.77-4.79]	0.164	0.59[0.18-1.88]	0.370
**Media exposure**				
Never reads newspapers ^R^	1		1	
Reads newspapers occasionally	1.10[0.47-2.60]	0.821	0.95[0.37-2.47]	0.917
Reads newspapers daily	0.74[0.33-1.68]	0.476	0.94[0.40-2.22]	0.888
**Number of women**	310		284	
